# A temporal-spectral dual-stream anti-noise bearing fault diagnosis model based on adaptive mode decomposition

**DOI:** 10.1038/s41598-026-55697-1

**Published:** 2026-06-08

**Authors:** Lingbo Li, Liyuan Ge, Jingyi Zhu, Weijun Jin, Yuheng Ren

**Affiliations:** 1 Digital Campus Construction Center, Zhejiang Technical Institute of Economics, Hangzhou, 310018 China; 2 Faculty of Art, University of Monash, Caulfield East, VIC 3145 Australia; 3 School of Automotive Technology, Zhejiang Technical Institute of Economics, Hangzhou, 310018 China; 4 China Mobile Communications Group Zhejiang Co, Ltd., Ningbo Branch, NingBo, 315100 China; 5 Digital Industry College, Jimei University, Fujian, 361199 China

**Keywords:** Bearing fault diagnosis, Background noise interference, Adaptive mode decomposition, Deep learning, Engineering, Mathematics and computing

## Abstract

In recent years, deep learning-based bearing fault diagnosis models have achieved remarkable performance under ideal experimental conditions. However, the accuracy and reliability of these models degrade significantly in real industrial scenarios where the acquired signals are often contaminated by strong background noise. Although a variety of anti-noise bearing fault diagnosis approaches have been developed, several limitations still remain. Most models directly take raw noisy signals as inputs, lacking an effective front-end noise suppression mechanism, which makes it difficult to sufficiently highlight fault-related information. Moreover, most models predominantly focus on the extraction and analysis of temporal features, neglecting the complementary fault characterization information embedded in other feature domains, thereby limiting the richness and discriminative power of feature representation. To address these issues, this paper proposes a temporal-spectral dual-stream anti-noise bearing fault diagnosis model based on adaptive mode decomposition. First, an adaptive mode decomposition module is designed to process raw noisy signals, aiming to suppress irrelevant noise components and enhance fault-related information, thereby providing a cleaner signal representation for subsequent diagnosis. Second, a temporal-spectral dual-stream bearing fault diagnosis framework is constructed to extract fault information from the same signal under different perspectives, aiming to enhance the richness and discriminative power of feature representation, thus the proposed model’s diagnostic performance under noise interference. Finally, extensive experimental results on two real-world cases demonstrate that, compared to current mainstream anti-noise bearing fault diagnosis models, the proposed approach achieves higher diagnostic accuracy under various noise intensities, sufficiently validating its effectiveness.

## Introduction

Rotating machinery constitutes the cornerstone of modern industrial systems and is widely deployed in critical fields such as wind power generation, rail transportation, and computer numerical control machine tools^1,2^. As the core transmission component of rotating machinery, the condition of bearings directly determines the reliability, safety, and service life of the entire equipment^3^. Statistics indicate that over 30% of rotating machinery failures can be traced back to bearing failures^4^. Sudden bearing faults may lead to unplanned shutdowns, production interruptions, and even catastrophic accidents, resulting in substantial economic losses and severe safety hazards^5–7^. Therefore, monitoring and accurately diagnosing the condition of bearings hold substantial theoretical and engineering value.

With the rapid development of the Internet and the continuous advancement of artificial intelligence theories, data-driven fault diagnosis methods have gradually become mainstream^8–13^ and have achieved excellent diagnostic performance under ideal experimental conditions^14,15^. Nevertheless, in practical industrial environments, acquired vibration signals are often severely corrupted by strong background noise, such as equipment coupled vibration, electromagnetic interference, and process-induced noise^16,17^. These interfering factors can significantly weaken fault-related characteristics, giving rise to the “feature inundation”phenomenon, ultimately leading to a notable decline in the accuracy and reliability of data-driven bearing fault diagnosis methods^18,19^.

Considering that the kurtogram can locate the optimal resonance frequency band sensitive to impact components by leveraging spectral kurtosis, effectively highlighting transient fault energy submerged in noise; and the recurrence plot can convert weak periodic fault impulses into regular recursive texture structures through phase space reconstruction, some studies convert noisy one-dimensional vibration signals into kurtograms or recurrence plots and then feed them into data-driven models for fault identification. For example, Udmale et al.^20^ proposed a novel intelligent fault diagnosis method for rotating machinery, which first reconstructs one-dimensional vibration signals into kurtograms and then inputs them into a convolutional neural network for fault classification. Nath et al.^21^ developed a fault diagnosis framework for rotating machinery that includes two parallel diagnostic branches. One branch takes fuzzy recurrence plots as input and uses a convolutional neural network to mine image texture fault features; the other branch takes unique frequency component streams as input and leverages a long short-term memory network to capture temporal correlation features of the signal. Udmale et al.^22^ proposed an improved random incremental extreme learning machine, which takes kurtograms converted from one-dimensional vibration signals as input and outputs the corresponding bearing fault categories. Shan et al.^23^ proposed the Mel-CNN, which converted vibration signals into Mel spectrograms to enhance the expressive power of fault information under noisy backgrounds and employed image recognition networks to mine fault features from an “acoustic fingerprint”perspective for bearing condition discrimination. Although such methods have achieved good fault diagnosis results, reconstructing one-dimensional vibration signals into two-dimensional images implies the subsequent need to use two-dimensional network architectures with a large number of parameters, which may increase computational complexity and impose high demands on hardware computing resources.

Therefore, other studies directly employ one-dimensional vibration signals to build anti-noise bearing fault diagnosis models. For instance, Udmale et al.^24^ extracted wavelet energy features from one-dimensional vibration signals, used a convolutional autoencoder to learn more distinguishable fault features from them, and combined with an extreme learning machine to achieve fault identification. Liu et al.^25^ constructed a multi-scale attention residual network to suppress noise interference in raw signals, while employing an attention weighting mechanism to guide the model to focus more on key regions related to fault features. Long et al.^26^ proposed a multi-model ensemble-based bearing fault diagnosis framework, which leverages the collaborative inference of multiple complementary models to effectively enhance the system’s diagnostic performance under noisy backgrounds. Zhao et al.^27^ introduced a deep residual shrinkage network (DRSN), which embeds a soft-thresholding shrinkage mechanism within the residual structures to adaptively filter out irrelevant noise information and highlight essential fault components. Chen et al.^28^ combined a multi-scale convolutional neural network (CNN) with a long short-term memory (LSTM) network to simultaneously capture local impact characteristics and long-term temporal dependencies of fault signals, thereby enhancing the model’s noise resistance. Hakim et al.^29^ mapped vibration signals into the frequency domain via Fast Fourier transform (FFT) and designed a lightweight one-dimensional CNN to mine discriminative spectral features, significantly mitigating the adverse impact of noise on model performance. Shi et al.^30^ proposed a residual denoising dynamic adaptive network (RDDAN), which combines DRSN with domain adaptation techniques to alleviate data distribution differences under varying operating conditions, improving the model’s fault identification capability under multiple noise and variable working conditions. Li et al.^31^ developed an adaptive multi-scale fully convolutional network (AMFCN), which enhances the adaptability to noise through a random sampling strategy while utilizing a multi-scale feature fusion mechanism to reduce the sensitivity of single-scale features to noise. Wang et al.^32^ proposed an adaptive denoising CNN (ADCNN) that dynamically suppresses background noise and strengthens fault-related information during feature learning, thereby achieving more reliable fault identification results. Tong et al.^33^ presented an improved deep residual shrinkage network (IDRSN) to alleviate signal distortion induced during the denoising process of DRSN, further strengthening denoising performance and enhancing the expressive power of fault features. Han et al.^34^ constructed a deep residual multi-scale convolutional neural network with an attention mechanism (Attention-MSCNN), which achieves precise focus on key fault information and effective suppression of irrelevant noise through the synergistic action of the attention mechanism and the multi-scale residual structure. Gao et al.^35^ designed a multi-time-scale attention module and an adaptive global-local denoising module based on DRSN to hierarchically model and process different noise components, enhancing the model’s adaptive capability in complex noise environments.

Although existing one-dimensional anti-noise bearing fault diagnosis models have made certain progress, several limitations still remain. First, most models directly take raw noisy vibration signals as input, where the mixed noise components may adversely affect the model’s feature extraction capability and diagnostic reliability. Second, most models primarily focus on time domain feature analysis. However, previous studies have demonstrated that frequency domain representations are more advantageous in highlighting periodic fault characteristics and suppressing random background noise. Third, most models rely on only a single feature domain for fault identification under noisy backgrounds, which can easily introduce modality bias (the time domain is sensitive to transient impact features but is vulnerable to random noise, whereas the frequency domain can stably reflect the spectral structure of fault characteristics but may lose time-varying phase information), thereby resulting in insufficient feature representation. To address the issues above, this paper proposes a temporal-spectral dual-stream anti-noise bearing fault diagnosis model based on adaptive mode decomposition. The main contributions of this work are summarized as follows:

1. An adaptive mode decomposition module is designed to decompose and reconstruct the raw noisy vibration signals at the front end, with the aim of suppressing irrelevant noise components and enhancing fault-related information, thereby providing high-quality input for subsequent diagnosis.
2. A temporal-spectral dual-stream bearing fault diagnosis framework is constructed. Through two parallel paths, it can simultaneously extract complementary fault information of the same signal from the time and frequency domains, so as to enhance the richness and discriminative power of feature representation, thus improving the model’s diagnostic performance under noise interference.
3. A serial local-global feature extraction extractor is developed for the time domain and frequency domain paths. By alternately and serially performing local feature extraction and global temporal modeling, it enables the feature information to be continuously refined and fused through an iterative process of“local *→* global *→* higher-level local *→* higher-level global”.
4. Extensive experiments are conducted on two real-world cases to evaluate the performance of the proposed model. The results demonstrate that, compared to current mainstream anti-noise bearing fault diagnosis models, the proposed approach achieves higher diagnostic accuracy under various noise intensities.

The remaining sections of this paper are arranged as follows: Section 2 systematically elaborates the overall framework and specific implementation procedures of the proposed model. Section 3 analyzes the fault diagnosis result of the proposed model under different noise intensities and compares it with current mainstream anti-noise models to validate its noise robustness. Section 4 discusses the limitations of this work and prospects the exploration direction of future work. Section 5 summarizes this work.

## Proposed approach

### Overall framework

The core idea of the proposed temporal-spectral dual-stream anti-noise bearing fault diagnosis model based on adaptive mode decomposition is“first purifying the raw noisy vibration signal, then performing multi-perspective deep-level fault diagnosis on the purified signal.”The proposed approach mainly consists of four stages: data preprocessing, noisy signal purification (adaptive mode decomposition module), multi-perspective feature extraction and fault diagnosis (temporal-spectral dual-stream bearing fault diagnosis framework), and model performance evaluation. The specific implementation steps for each stage are as follows:

*Data preprocessing:* First, the raw vibration sequence $R^{T}$ (time domain) collected by sensors is segmented using a sliding window into *N* equal-length time signals with a length of 1024, denoted as $S_{T}=\{s_{1},s_{2},\ldots ,s_{N}\}$. Next, to simulate industrial scenarios under different noise conditions, noise with varying intensities is added to $S_{T}$, thereby constructing noisy signals under different signal-to-noise ratio conditions. Subsequently, these noisy signals are standardized to eliminate scale and amplitude differences. Finally, the standardized signals are randomly split into training and validation sets with a ratio of 7.5 : 2.5. This process is mathematically expressed as:

1 $$\begin{aligned} & \begin{aligned}&s_{i}^{st}=\frac{s_{i}^{n}-\mu _{i}^{n}}{\sigma _{i}^{n}}, \quad i=1,2,\ldots ,N, \\&S_{T}^{st}=\{s_{1}^{st},s_{2}^{st},\ldots ,s_{N}^{st}\}. \end{aligned} \end{aligned}$$

2 $$\begin{aligned} & S_{T}^{st}=D_{train}\cup D_{val},\quad |D_{train}|:|D_{val}|=7.5:2.5,\quad D_{train}\cap D_{val}=\varnothing . \end{aligned}$$

where $s_{i}^{n}$ and $s_{i}^{st}$ denote the noisy signal and the standardized signal, respectively. $\mu _i^{n}$ and $\sigma _i^{n}$ represent the mean and standard deviation of $s_i^{n}$, respectively. $D_{train}$ and $D_{val}$ represent the training set and validation set.

*Noisy signal purification:* Each sample in $D_{train}=\{s_{1}^{st},s_{2}^{st},\ldots ,s_{0.75N}^{st}\}$ is sequentially fed into the adaptive mode decomposition module, which can select the modal components that best characterize fault-related information according to a predefined criterion and reconstruct them to obtain the purified time domain signal $\tilde{D}_{train}^{time}=\{t_{1},t_{2},\ldots ,t_{0.75N}\}$. Fast Fourier transform (FFT) is further applied to $\tilde{D}_{train}^{time}$ to obtain the corresponding frequency domain representation $\tilde{D}_{train}^{fre}=\{fre_{1},fre_{2},\ldots ,fre_{0.75N}\}$.

3 $$\begin{aligned} \tilde{D}_{train}^{time}=\psi _{AMDM}(D_{train};\theta _{AMDM}),\qquad \tilde{D}_{train}^{fre}=FFT(\tilde{D}_{train}^{time}). \end{aligned}$$

where $\psi _{AMDM}(\cdot )$ denotes the proposed adaptive mode decomposition module with learnable parameters $\theta _{AMDM}$.

*Multi-perspective feature extraction and fault diagnosis:* Each sample from $\tilde{D}_{train}^{time}$ and $\tilde{D}_{train}^{fre}$ is fed into the temporal-spectral dual-stream bearing fault diagnosis framework to obtain high-dimensional feature representations of the same signal from different perspectives. Subsequently, these two sets of feature vectors are fused and passed through a fully connected layer and a Softmax activation function to output the predicted probability distribution over fault categories.

*Model performance evaluation:* Each sample in $D_{val}=\{s_{0.75N+1}^{st},s_{0.75N+2}^{st},\ldots ,s_{N}^{st}\}$ is sequentially fed into the trained adaptive mode decomposition module and the temporal-spectral dual-stream bearing fault diagnosis framework to calculate the fault recognition accuracy, aiming to evaluate the noise robustness and generalization capability of the proposed model.

### Adaptive mode decomposition module

Given that the noise components mixed in the raw vibration signals can act as“spurious patterns”that interfere with the model’s feature learning, this paper proposes an adaptive mode decomposition module. This module can suppress irrelevant noise and enhance fault-related information through a three-step strategy of “decomposition-evaluation-reconstruction.”

Specifically, the proposed module adopts variational mode decomposition (VMD) as the core decomposer to adaptively decompose the input signal into *K* intrinsic mode functions (IMFs) with specific center frequencies $\{\omega _k\}$, denoted as $u_k(k=1,2,\ldots ,K)$. The variational problem of VMD aims to find a set of modes $\{u_k\}$ and their corresponding center frequencies $\{\omega _k\}$ that minimize the sum of the estimated bandwidths of all modes while satisfying the constraint that the sum of all modes equals the original signal. The mathematical expression is as follows:

4 $$\begin{aligned} \begin{aligned}&\text {s.t.}\ \sum _{k=1}^{K}u_{k} = s_i^{st},\quad i=1,2,\ldots ,0.75N, \\&\min _{\{u_k\},\{\omega _k\}} \left\{ \sum _{k=1}^{K} \left\| \partial _t\left[ \left( \delta (t)+\frac{j}{\pi t}\right) *u_k(t) \right] e^{-j\omega _k t} \right\| _2^2 \right\} . \end{aligned} \end{aligned}$$

Here, $s_i^{st}$ represents the *i*-th sample in the training set; $\partial _t$ denotes the partial derivative with respect to time; *δ (t)* is the Dirac delta function; * represents convolution operation; and *j* is the imaginary unit. By introducing the quadratic penalty term *α* and the Lagrange multiplier *λ*, the above constrained optimization problem can be converted into an unconstrained one and solved iteratively via the alternating direction method of multipliers (ADMM). In this article, *α* is set to 2000, and the maximum number of ADMM iterations is set to 500.

However, traditional VMD requires pre-setting the value of *K*. If *K* is inappropriate, it may lead to under-decomposition or over-decomposition. Considering that the envelope spectrum peak saliency index (ESPS) can measure the prominence of the peak at the fault characteristic frequency in the envelope spectrum relative to the average energy, effectively reflecting the retention effect of mode decomposition on fault-related components. Compared with spectral kurtosis or entropy-based measures, ESPS focuses more on the periodic modulation information at fault characteristic frequencies (which is more consistent with the core objective of rolling bearing fault diagnosis) and comprehensively considers the contributions of all decomposed modes, avoiding the judgment bias caused by the dominance of a single mode. Therefore, the ESPS is introduced as an adaptive criterion. Specifically, for each signal $s_i^{st}$ to be decomposed, the optimal number of modes is searched within the candidate range $K\in [K_{\min },K_{\max }]$, where $K_{\min }$ and $K_{\max }$ are set to 2 and 10, respectively. For each candidate *K*, VMD is first performed to obtain *K* IMFs. Next, the envelope spectrum $E_k(f)$ of each IMF $u_k$ is calculated, and the overall ESPS value corresponding to that *K* value is then computed. The formula is as follows:

5 $$\begin{aligned} ESPS(K)=\frac{1}{K}\sum _{k=1}^{K} \left( \frac{\max _{f\in F_{fault}}E_k(f)}{\textrm{mean}(E_k(f))} \right) . \end{aligned}$$

where $F_{fault}$ is the theoretical fault characteristic frequency calculated based on bearing geometric parameters. Finally, the *K* value that maximizes *ESPS*(*K*) is selected as the optimal decomposition number, which encourages the decomposed modal set to maximize the aggregation periodic impact energy related to faults.

It should be noted that among the obtained *K* IMFs, not all modes are equally important (noise-dominant modes may obscure fault information). Therefore, a weighted reconstruction strategy needs to be designed to assign a reconstruction weight $\beta _{k}$ to each IMF. Since kurtosis is highly sensitive to local impact components in the signal and the Pearson correlation coefficient can measure the linear correlation between IMFs and the signal $s_i^{st}$ to preserve components associated with the structure of $s_i^{st}$, this paper uses the normalized product of kurtosis $Ku_{k}$ and Pearson correlation coefficient $\rho _{k}$ as the final weight $\beta _{i,k}$ to simultaneously consider the impact strength and contribution to the signal $s_i^{st}$. The calculation formula is as follows:

6 $$\begin{aligned} & \begin{aligned}&Ku_{i,k}= \frac{\frac{1}{L}\sum _{\tau =1}^{L}\left( u_{i,k}(\tau )-\bar{u}_{i,k}\right) ^4}{\left( \frac{1}{L}\sum _{\tau =1}^{L}\left( u_{i,k}(\tau )-\bar{u}_{i,k}\right) ^2\right) ^2},\qquad \rho _{i,k}= \frac{\sum _{\tau =1}^{L}\left( u_{i,k}(\tau )-\bar{u}_{i,k}\right) \left( s_i^{st}(\tau )-\bar{s}_i^{st}\right) }{\sqrt{\sum _{\tau =1}^{L}\left( u_{i,k}(\tau )-\bar{u}_{i,k}\right) ^2 \sum _{\tau =1}^{L}\left( s_i^{st}(\tau )-\bar{s}_i^{st}\right) ^2}}, \\&\tau = \{1, 2, \ldots , L\},\qquad L=1024. \end{aligned} \end{aligned}$$

7 $$\begin{aligned} & \tilde{K}u_{i,k}=\frac{K u_{i,k}}{\max _j K u_{i,j}},\qquad \tilde{\rho }_{i,k}=\frac{|\rho _{i,k}|}{\max _j|\rho _{i,j}|}. \end{aligned}$$

8 $$\begin{aligned} & \beta _{i,k}= \frac{\tilde{K}u_{i,k}\cdot \tilde{\rho }_{i,k}}{\sum _{j=1}^{K}\tilde{K}u_{i,j}\cdot \tilde{\rho }_{i,j}}. \end{aligned}$$

where $\bar{u}_{i,k}$ is the mean of the *k*-th IMF for the *i*-th sample, and *L* is the sample length. Finally, the selected IMFs are linearly weighted using $\beta _{i,k}$ to obtain the purified time domain signal representation $t_i$:

9 $$\begin{aligned} t_i=\sum _{k=1}^{K}\beta _{i,k}\,u_{i,k},\quad i=1,2,\ldots ,0.75N. \end{aligned}$$

The reconstructed signal $t_i$ enhances fault-related impact components while preserving the main structure of the signal $s_i^{st}$ and suppressing irrelevant random noise.

### Temporal-spectral dual-stream bearing fault diagnosis framework

**Fig. 1:**
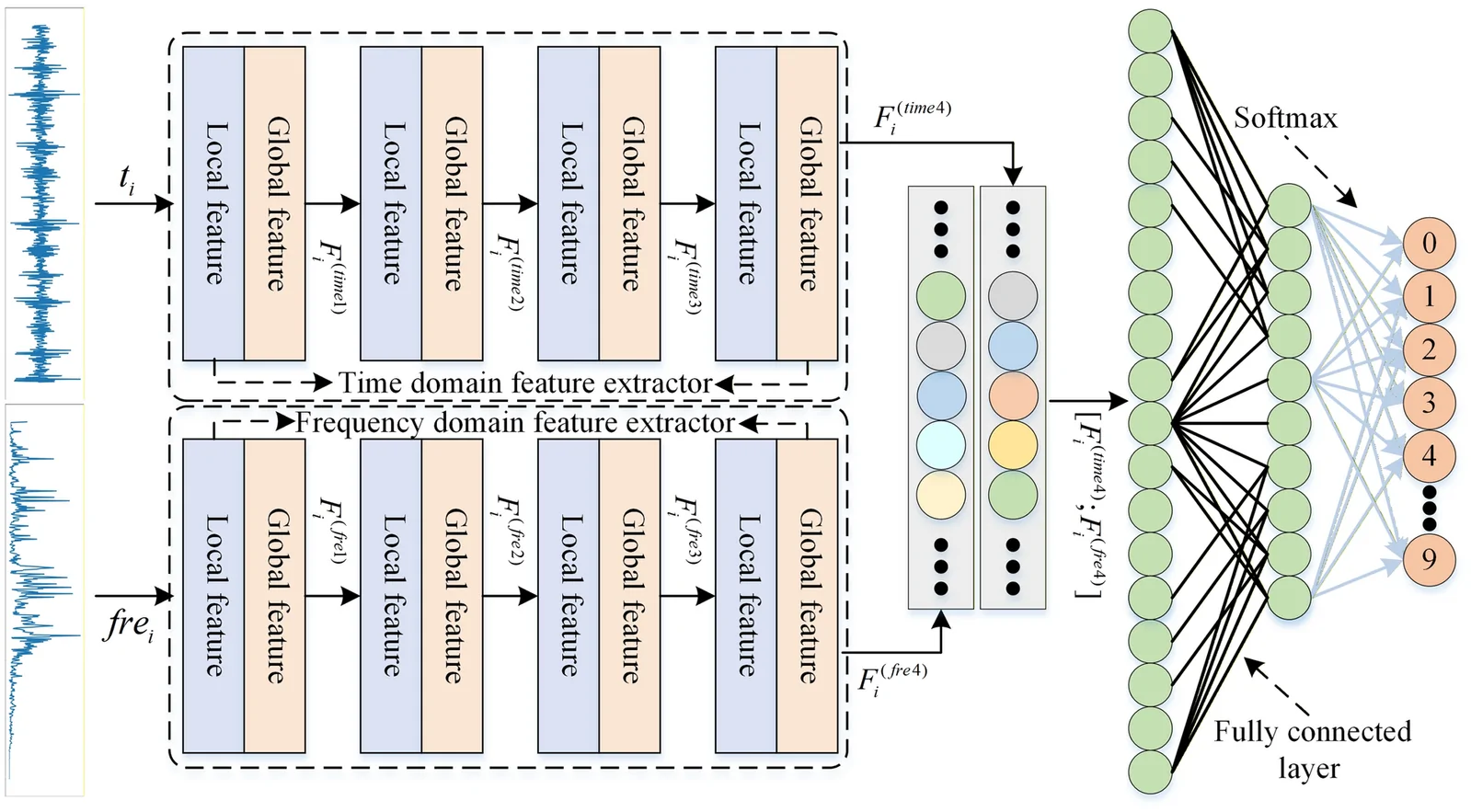
The overall architecture of the temporal-spectral dual-stream bearing fault diagnosis framework.

Although the proposed adaptive mode decomposition module can preliminarily suppress background noise at the signal level, residual noise under strong noise environments may still cause the model to learn unstable features. Meanwhile, single-domain feature representations of the signal may suffer from information loss or modal bias. To address these issues, this paper constructs a temporal-spectral dual-stream bearing fault diagnosis framework (as shown in Fig. 1). It can mine the“two mutually complementary representations of the homologous signal”through parallel dual paths and fuse them at the feature level, thereby enhancing the fault identification accuracy and robustness of the proposed model under strong noise backgrounds.

Specifically, given the purified time domain signal $\tilde{D}_{train}^{time}=\{t_{1},t_{2},\ldots ,t_{0.75N}\}$ and its corresponding frequency domain representation $\tilde{D}_{train}^{fre}=\{fre_{1},fre_{2},\ldots ,fre_{0.75N}\}$, define the time domain path feature extractor and the frequency domain path feature extractor as $\phi _t(\cdot ;\theta _t)$ and $\phi _f(\cdot ;\theta _f)$, respectively. The outputs of the dual paths are $F_i^{(time4)}$ and $F_i^{(fre4)}$, which are concatenated along the channel dimension and fed into the fully connected layer and Softmax activation function to complete the bearing state identification for the current sample. The formulas are as follows:

10 $$\begin{aligned} & F_i^{(time4)}=\phi _t(t_i;\theta _t),\qquad F_i^{(fre4)}=\phi _f(fre_i;\theta _f). \end{aligned}$$

11 $$\begin{aligned} & F_i^{(\mathrm {t-f})}=\left[ \,F_i^{(time4)};\,F_i^{(fre4)}\,\right] ,\quad i=1,2,\ldots ,0.75N. \end{aligned}$$

12 $$\begin{aligned} & \hat{y}=\textrm{Softmax}\left( W_c\,F_i^{(\mathrm {t-f})}+b_c\right) . \end{aligned}$$

where $\theta _t$ and $\theta _f$ are learnable parameters of the time domain and frequency domain path feature extractors, respectively. $F_i^{(time4)}$ and $F_i^{(fre4)}$ represent the high-dimensional feature representations of the *i*-th signal in the time and frequency domains. $[\cdot ;\cdot ]$ denotes the concatenation operation along the channel dimension. $\hat{y}$ is the predicted probability distribution, and $W_c$ and $b_c$ represent the weight matrix and bias of the fully connected layer, respectively. This design can describe fault information from two perspectives“time-varying morphology”and“spectral-structure stability” making the model less prone to“dropping critical features”under strong noise.

**Fig. 2:**
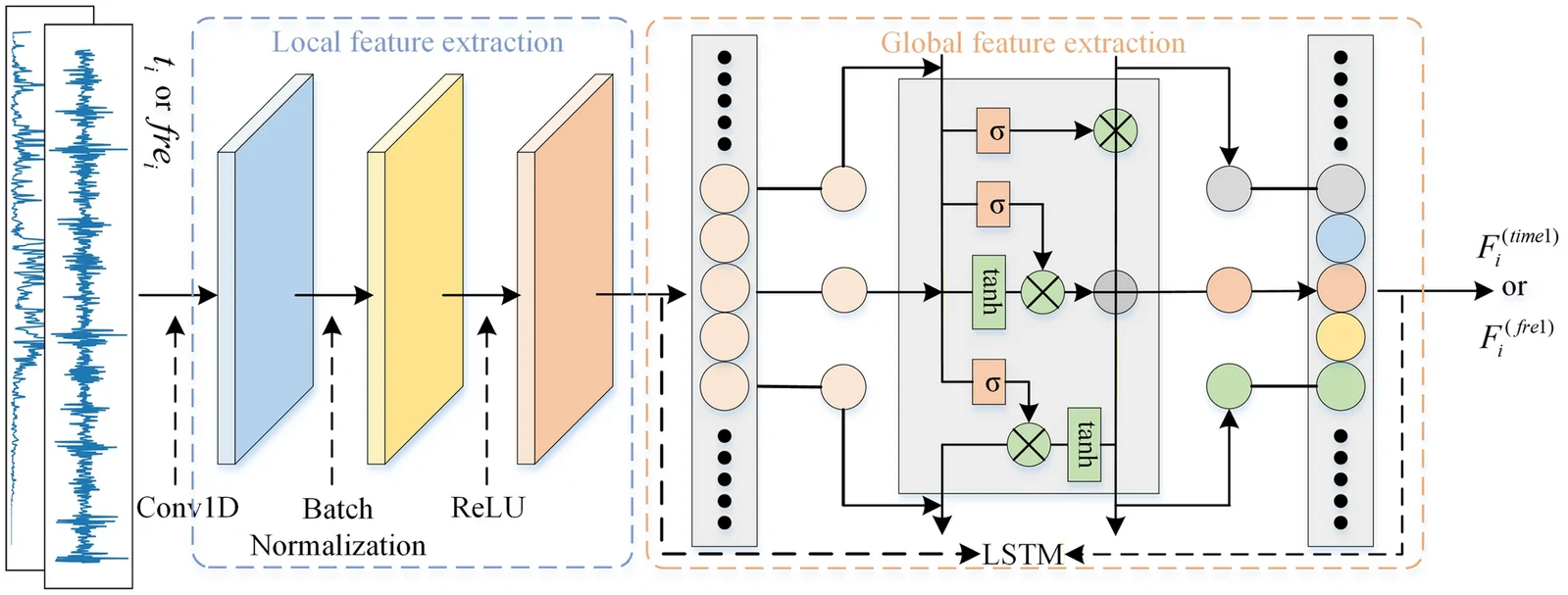
The specific structure of the serial local-global feature extractor.

Furthermore, considering that both time domain sequences and frequency domain sequences are essentially one-dimensional sequences, they not only contain rich local detailed patterns (e.g., transient impulses, local harmonic peaks, sidebands) but also exhibit long range dependencies (e.g., periodicity of impulses, cross-band coupling, long range correlations). Therefore, this paper designs a novel serial local-global feature extractor for each path (as shown in Fig. 2). Its core idea is to alternately and serially perform local feature extraction and global temporal modeling on the current time/frequency domain representation, allowing information to be continuously refined and fused in an iterative cycle of “local *→* global *→* more abstract local *→* more macroscopic global.” This extractor consists of four cascaded local-global blocks, and its computational process is as follows:

13 $$\begin{aligned} \begin{aligned}&F_i^{(time1)}=\textrm{LSTM}\!\left( \textrm{ReLU}\!\left( \textrm{BN}\!\left( \textrm{Conv1D}(t_i)\right) \right) \right) ,\quad F_i^{(fre1)}=\textrm{LSTM}\!\left( \textrm{ReLU}\!\left( \textrm{BN}\!\left( \textrm{Conv1D}(fre_i)\right) \right) \right) , \\&\cdots \cdots ,\\&F_i^{(time4)}=\textrm{LSTM}\!\left( \textrm{ReLU}\!\left( \textrm{BN}\!\left( \textrm{Conv1D}(F_i^{(time3)})\right) \right) \right) ,\quad F_i^{(fre4)}=\textrm{LSTM}\!\left( \textrm{ReLU}\!\left( \textrm{BN}\!\left( \textrm{Conv1D}(F_i^{(fre3)})\right) \right) \right) . \end{aligned} \end{aligned}$$

This design achieves a layer-by-layer deepening of understanding from micro-impulse morphology to macro-temporal patterns for time or frequency representations. It enables the model to not only accurately capture local transient characteristics of faults but also effectively model their long range dependencies across time scales.

## Experimental results and analysis

All experiments in this section were conducted on a unified computing platform. The operating system was Windows 11, and the hardware configuration consisted of a 12th Gen $\text {Intel}^{\circledR } \text {Core}^{\textrm{TM}}$ i5 processor and an NVIDIA RTX 4090 GPU. The algorithm was implemented in Python 3.9.0 (URL: https://www.python.org/) , with development and training carried out using the PyCharm 2023.3.2 (URL: https://www.jetbrains.com/pycharm/) integrated development environment and the PyTorch 2.1.0 (URL: https://pytorch.org/) deep learning framework. During model training, the Adam optimizer was used with an initial learning rate of 0.001, and a cosine annealing scheduler was employed to dynamically adjust the learning rate, with the minimum learning rate set to $1 \times 10^{-6}$. The batch size was set to 64, and the maximum number of training epochs was set to 200. To prevent overfitting, an early stopping strategy was applied: training was terminated if the loss on the validation set did not decrease for 20 consecutive epochs. No additional data augmentation was applied, so as to preserve the purity of the evaluation of noise effects. All experiments were repeated 10 times (for each dataset, 10 independent random splits were performed, and in each run, the samples were divided into mutually exclusive training and validation sets at a ratio of 7.5:2.5), and the final results are reported as mean ± standard deviation to more comprehensively reflect the robustness and stability of the proposed method.

### Simulation of noisy environments

The vibration signals in actual industrial settings are often affected by multiple random factors such as mechanical coupling, process fluctuations, and electromagnetic interference, resulting in noise components that exhibit significant randomness and uncontrollability. Considering that white Gaussian noise has a uniform power spectral density and is widely used as a classical model for random noise interference, in this study, additive white Gaussian noise (AWGN) is employed to simulate complex background noise environments. Specifically, by adding AWGN with different power levels to the original clean vibration signals, five noisy datasets with signal-to-noise ratios (SNRs) of -2 dB, -4 dB, -6 dB, -8 dB, and -10 dB are constructed to comprehensively evaluate the anti-noise performance of the proposed model. The SNR is defined as the logarithmic ratio of the average power of the original clean signal to the average power of the added noise, which can be calculated as follows:

14 $$\begin{aligned} SNR = 10 \log _{10} \left( \frac{P_{\text {signal}}}{P_{\text {noise}}} \right) (dB). \end{aligned}$$

Here, $P_{\text {signal}}$ and $P_{\text {noise}}$ denote the average power of the original clean vibration signal and the added AWGN, respectively. A lower SNR value indicates a stronger noise component, making characteristics such as fault impacts and modulation sidebands more susceptible to being obscured.

### Dataset description

To validate the effectiveness and generalization capability of the proposed temporal-spectral dual-stream anti-noise bearing fault diagnosis model based on adaptive mode decomposition under complex noise conditions, extensive experiments were conducted and evaluated on two public bearing datasets: CWRU and MFPT.

**CWRU Dataset:** The dataset encompasses four bearing states: normal, inner race fault, outer race fault, and rolling element fault. Each fault type includes three levels of damage diameter (0.007, 0.014, and 0.021 inches), resulting in a total of 10 fault categories that exhibit differences in both“fault location”and “fault severity”. In this study, vibration signals collected by the drive-end accelerometer under a motor load of 0 hp and a sampling frequency of 12 kHz are used for experiments. After applying a sliding window segmentation to the raw vibration signals, the segment length of all samples is uniformly set to 1024, and each category contains 300 training samples and 100 test samples. Detailed information is summarized in Table 1.

**Table 1:** Detailed description of the CWRU dataset used in the experiments.

Labels	Fault location	Fault diameter	Sample length	Training number	Test number
RF_007	Roller fault	0.007 inch	1024	300	100
RF_014		0.014 inch	1024	300	100
RF_021		0.021 inch	1024	300	100
IR_007	Inner race	0.007 inch	1024	300	100
IR_014		0.014 inch	1024	300	100
IR_021		0.021 inch	1024	300	100
OR_007	Outer race	0.007 inch	1024	300	100
OR_014		0.014 inch	1024	300	100
OR_021		0.021 inch	1024	300	100
Normal	Normal	-	1024	300	100

**MFPT Dataset:** The MFPT dataset is more diverse than CWRU and contains a total of 23 categories. In this study, 9 categories are selected from the MFPT dataset for experiments, including 4 inner race fault categories, 4 outer race fault categories, and 1 normal category. Specifically, the inner race fault data correspond to load conditions of 0, 50, 150, and 300 lbs, while the outer race fault data correspond to load conditions of 25, 50, 150, and 300 lbs; the sampling frequency for both fault types is 48828 Hz. The normal state data are acquired under a load of 270 lbs with a sampling frequency of 97,656 Hz. Similarly, after applying a sliding window segmentation to the raw vibration signals, the segment length of all samples is uniformly set to 1024, and each category contains 300 training samples and 100 test samples. Detailed information is presented in Table 2.

**Table 2:** Detailed description of the MFPT dataset used in the experiments.

Labels	Fault location	Sample frequeney	Load	Sample length	Training number	Test number
IR_000	Inner race	48828 Hz	000 Lbs	1024	300	100
IR_050		48828 Hz	050 Lbs	1024	300	100
IR_150		48828 Hz	150 Lbs	1024	300	100
IR_300		48828 Hz	300 Lbs	1024	300	100
OR_025	Outer race	48828 Hz	025 Lbs	1024	300	100
OR_050		48828 Hz	050 Lbs	1024	300	100
OR_150		48828 Hz	150 Lbs	1024	300	100
OR_300		48828 Hz	300 Lbs	1024	300	100
Normal	Normal	97656 Hz	270 Lbs	1024	300	100

It should be noted that no cross-condition validation was performed in this study, meaning that for each fault category, the samples of the training and test sets are from the same operating condition. Therefore, the generalization capability discussed in this study mainly refers to the robustness of the proposed model under different noise intensities, rather than its transferability across different operating conditions.

### Diagnostic results under different noise intensities

**Table 3:** Accuracy, Precision, Recall, and F1-Score of the proposed model on the CWRU and MFPT datasets under different SNR conditions (-2 dB to -10 dB).

Dataset	SNR	Accuracy (%)	Precision (%)	Recall (%)	F1-Score (%)
CWRU	-2dB	99.17±0.41%	99.23±0.38%	99.31±0.42%	99.14±0.47%
	-4dB	97.40±0.58%	97.52±0.62%	97.61±0.55%	97.43±0.61%
	-6dB	91.26±0.89%	91.18±0.93%	91.47±0.87%	90.89±0.96%
	-8dB	87.93±1.14%	87.81±1.21%	88.12±1.09%	87.54±1.27%
	-10dB	82.14±1.46%	82.07±1.52%	82.38±1.41%	81.79±1.58%
MFPT	-2dB	96.07±0.53%	96.14±0.49%	96.28±0.57%	96.01±0.54%
	-4dB	93.14±0.74%	93.27±0.71%	93.42±0.68%	93.13±0.78%
	-6dB	87.56±1.12%	87.49±1.08%	87.71±1.15%	87.28±1.21%
	-8dB	82.29±1.38%	82.16±1.44%	82.54±1.31%	81.83±1.47%
	-10dB	78.67±1.69%	78.52±1.73%	78.91±1.64%	78.18±1.79%

Table 3 presents the fault identification accuracy, precision, recall, and F1-score (all with standard deviations) of the proposed model on both the CWRU and MFPT datasets under different SNR conditions. Overall, results from both datasets show a monotonic decrease in accuracy, precision, recall, and F1-score as the noise intensifies (SNR decreasing from -2 dB to -10 dB). This is because as the noise intensity gradually increases, the amplitude contrast of fault impacts in the time domain is progressively weakened, while the periodic spectral components related to bearing fault characteristic frequencies in the frequency domain are gradually masked. The continuous degradation of these two types of fault discriminative information leads to a gradual decline in the diagnostic performance of the proposed model. Specifically, on the CWRU dataset, the proposed model achieves 99.17±0.41%, 99.23±0.38%, 99.31±0.42%, and 99.14±0.47% in Accuracy, Precision, Recall, and F1-score, respectively, at -2 dB, and 97.40±0.58%, 97.52±0.62%, 97.61±0.55%, and 97.43±0.61%, respectively, at -4 dB, demonstrating “near-saturated”recognition performance. As noise increases further to -6 dB, -8 dB, and -10 dB, accuracy decreases to 91.26±0.89%, 87.93±1.14%, and 82.14±1.46%, while Precision, Recall, and F1-score show a similar downward trend, indicating that strong noise not only reduces the overall classification correctness but also weakens the balanced discrimination capability across categories. In contrast, on the MFPT dataset, all four metrics are generally lower at equivalent SNRs (e.g., 78.67±1.69% in Accuracy, 78.52±1.73% in Precision, 78.91±1.64% in Recall, and 78.18±1.79% in F1-score at -10 dB) and decline more rapidly (a greater drop from -2 dB to -10 dB). This paper considers that this phenomenon may be attributed to the MFPT dataset having finer-grained categories and larger load span. Intense noise not only attenuates fault information but also amplifies the distribution overlap among different loads/states, thereby increasing the probability of misclassification. Despite this, the proposed model maintains relatively stable performance even in the strong noise regimes of both datasets, indicating that the adaptive mode decomposition module effectively enhances input signal quality, while the temporal-spectral dual-stream bearing fault diagnosis framework can bolster robustness to residual noise through multi-perspective feature fusion. More importantly, the consistently low standard deviations across all four metrics suggest that the proposed method maintains good statistical stability under different noise levels. This result also helps position the proposed method more clearly: rather than pursuing the highest score at a specific SNR only, it is designed to preserve stable and balanced diagnostic performance over a wide noise range, especially under severe noise conditions.

**Fig. 3:**
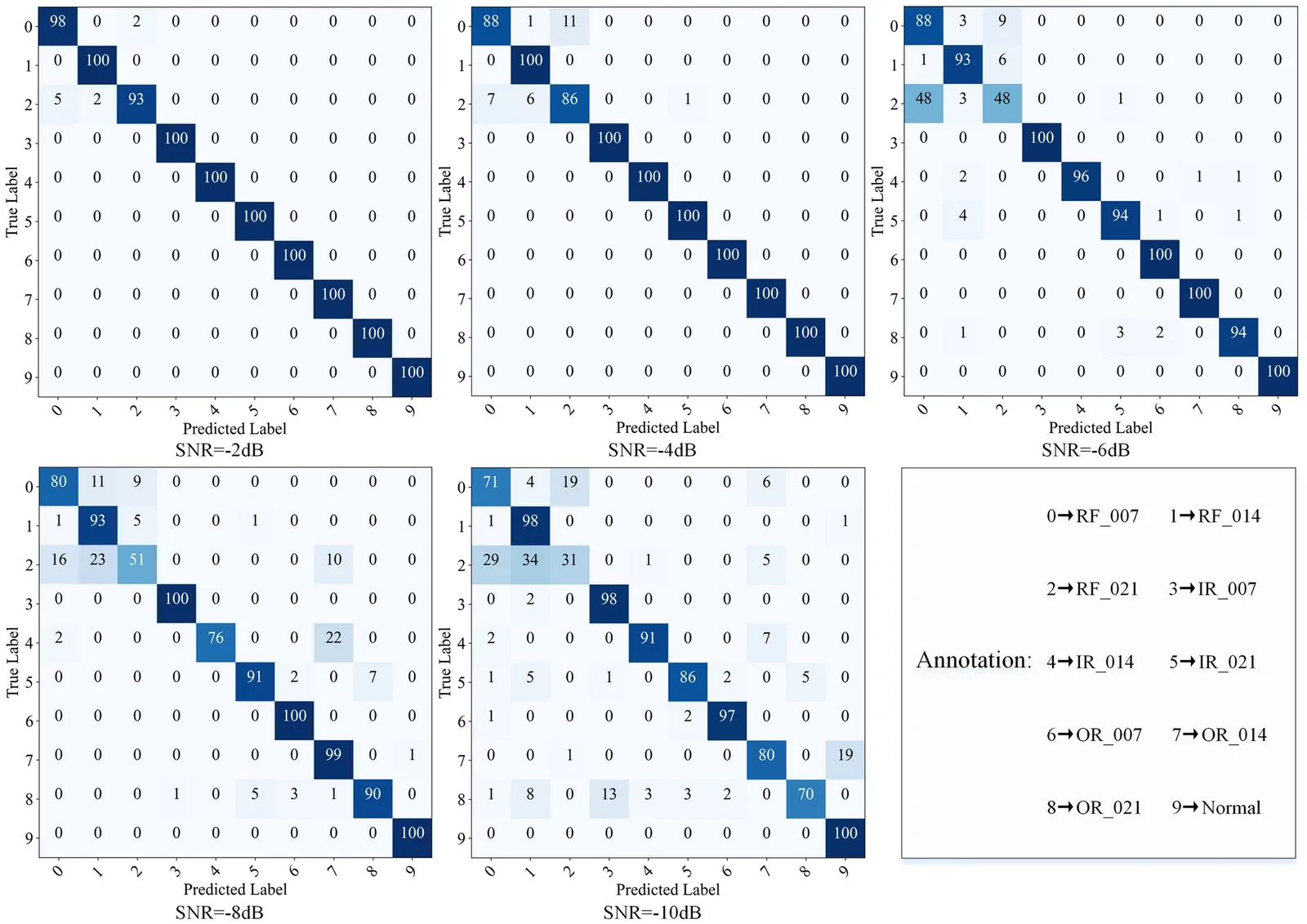
Confusion matrices for accuracy of the proposed model on the CWRU dataset under different SNR conditions (-2 dB to -10 dB).

**Fig. 4:**
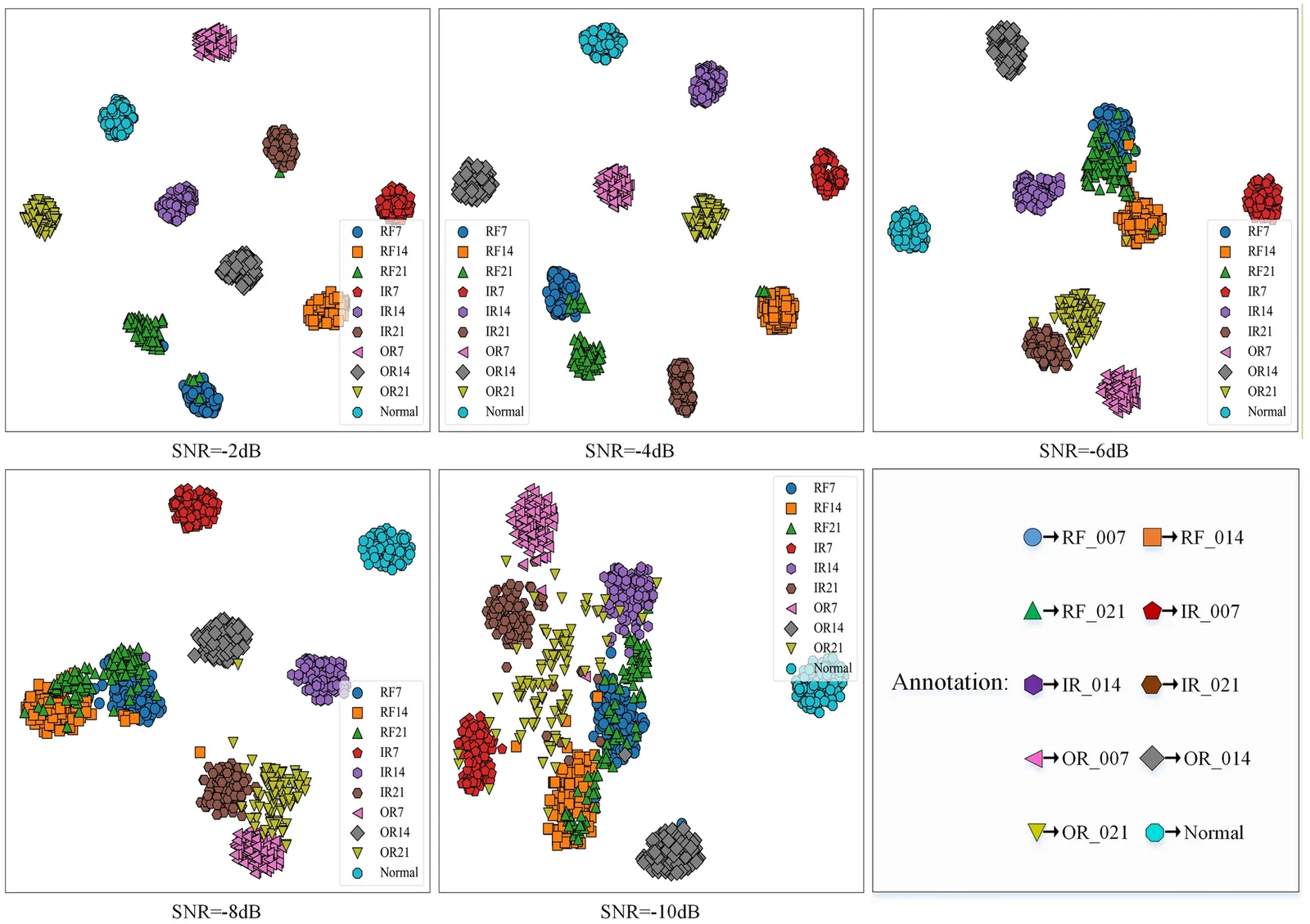
t-SNE visualization results of the proposed model on the CWRU dataset under different SNR conditions (-2 dB to -10 dB).

To delve deeper into the diagnostic mechanism and performance boundaries of the proposed model, Figs. 3 and 4 respectively display the accuracy confusion matrices and the corresponding t-SNE feature dimensionality reduction plots on the CWRU dataset across the full noise range from -2 dB to -10 dB. It can be observed that, on the CWRU dataset, the evolution patterns of the accuracy confusion matrices and t-SNE plots are consistent: increasing noise gradually shrinks the inter-class margins, but misclassifications are primarily concentrated among different damage sizes within the same fault type. The underlying reason lies in the fact that different fault locations (rolling element, inner race, and outer race) exhibit relatively distinct and stable impulse repetition patterns and spectral distribution characteristics. In contrast, the differences among different damage sizes under the same fault type are mainly reflected in subtle amplitude variations and local waveform features, which are more susceptible to noise interference. Specifically, at -2 dB, the confusion matrix is almost perfectly diagonal, t-SNE shows compact and well-separated clusters, with clear boundaries between the healthy state and various fault classes. At -4 dB, only minor fine-grained confusion occurs (slight mutual misclassification among different sizes of the same class), and t-SNE reveals reduced distances between clusters of similar categories while overall separation is maintained. From -6 dB onwards, off-diagonal entries become noticeably more frequent, with misclassifications mainly occurring between adjacent sizes within each of the RF/IR/OR fault types; t-SNE manifests as partial boundary contact or local overlap between some neighboring clusters, though the overall cluster structure remains discernible. At -8 dB, intra-class confusion intensifies further, accompanied by a trend of limited misclassification across different fault locations; t-SNE shows significantly reduced margins and expanded overlapping regions among multiple clusters. At -10 dB, confusion is most pronounced, yet errors are still predominantly among“semantically similar categories”rather than being randomly diffused; t-SNE indicates heavier cluster mixing but not complete collapse.

**Fig. 5:**
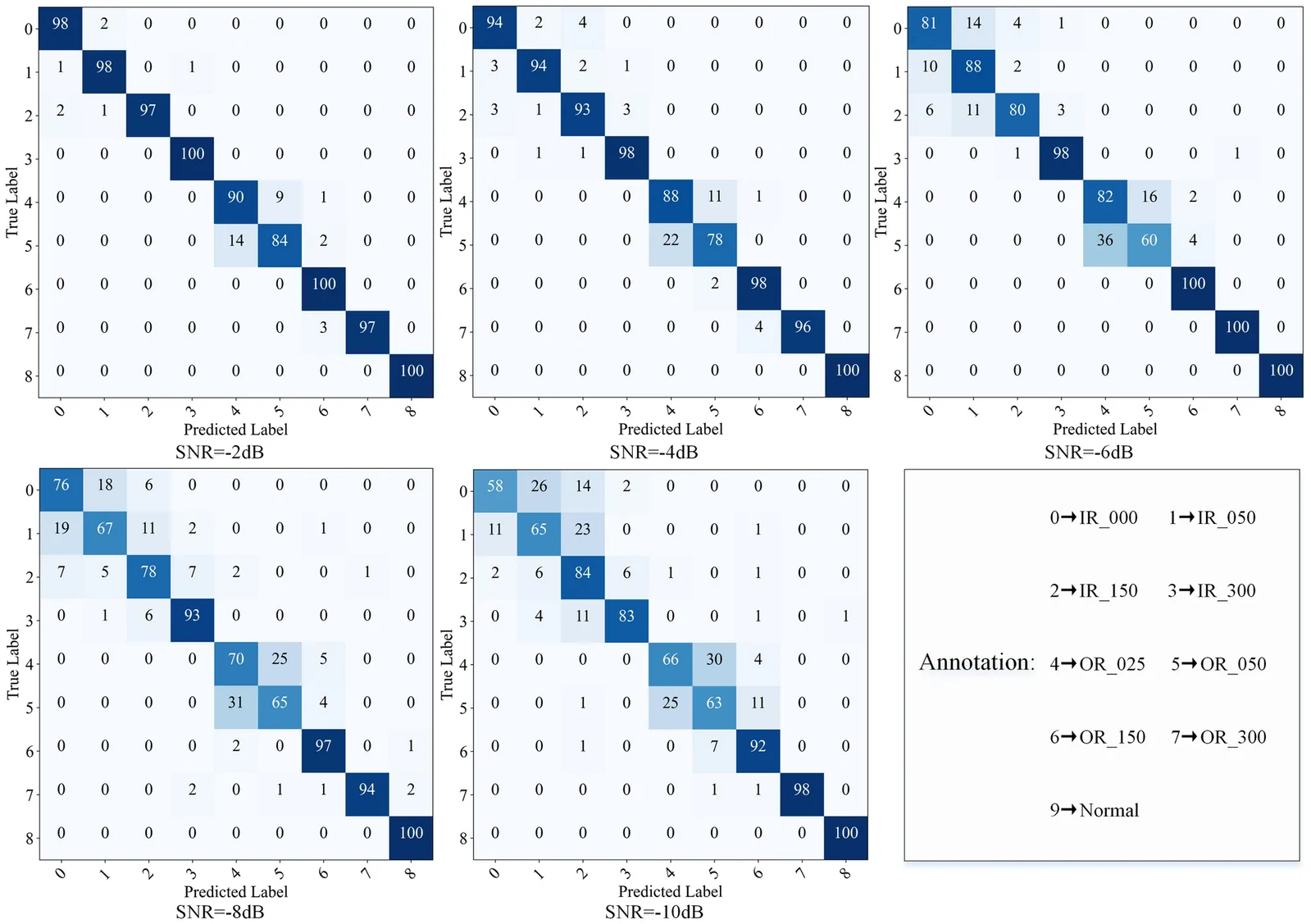
Confusion matrices for accuracy of the proposed model on the MFPT dataset under different SNR conditions (– 2 dB to – 10 dB).

**Fig. 6:**
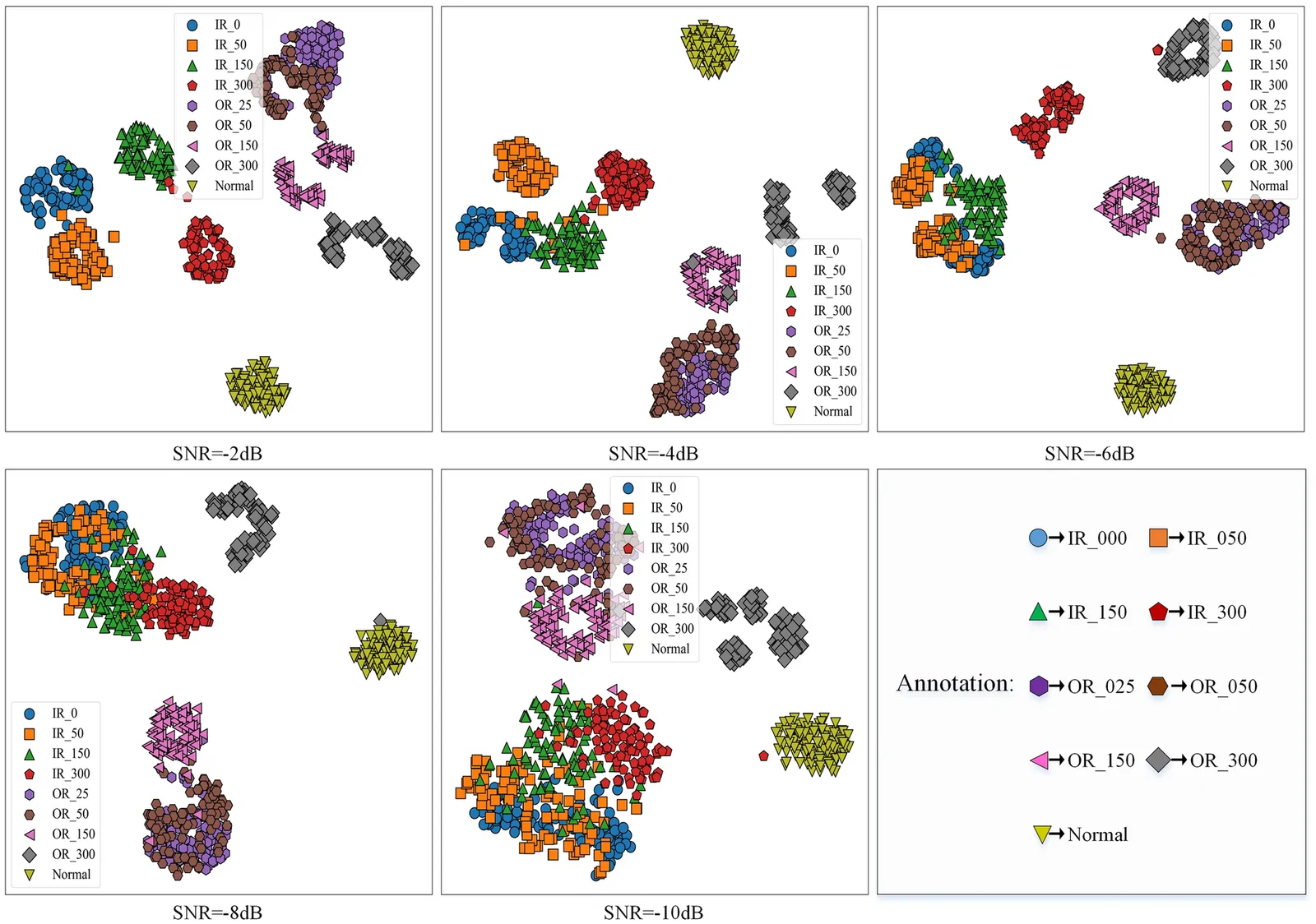
t-SNE visualization results of the proposed model on the MFPT dataset under different SNR conditions (– 2 dB to – 10 dB).

Similarly, Figs. 5 and 6 respectively present the accuracy confusion matrices and t-SNE plots on the MFPT dataset across the same noise range. Due to the finer categories and wider operational condition span of MFPT, inter-class proximity and mixing appear earlier under equivalent noise levels. Specifically, at -2 dB, the confusion matrix is predominantly diagonal overall, with the healthy class remaining stable, but slight mutual misclassification is already visible among adjacent load/state categories; t-SNE shows clear clusters for most classes, though distances between neighboring category clusters are relatively close. At -4 dB, misclassifications between adjacent categories increase, with errors spreading directionally according to“adjacent load relationships”; t-SNE shows boundary contact between adjacent clusters. From -6 dB onwards, more pronounced“intra-group aliasing”forms (mutual confusion among the same fault type under different loads), and t-SNE reveals interwoven point clouds from multiple similar categories forming larger patches. However, the healthy class typically remains relatively isolated, indicating the model’s continued reliability in coarse-grained“healthy/faulty” discrimination. At – 8 dB and – 10 dB, the scope of aliasing continues to expand, fine-grained category boundaries significantly weaken, and t-SNE shows increased mixing areas. Nevertheless, the healthy class and some fault categories maintain relatively concentrated distributions, suggesting that although the proposed model may struggle to stably distinguish fine-grained operational differences under extreme noise, it can still preserve a structured feature space to support its overall diagnostic performance in strong noise conditions.

### Diagnostic performance comparison with current mainstream anti-noise models

**Fig. 7:**
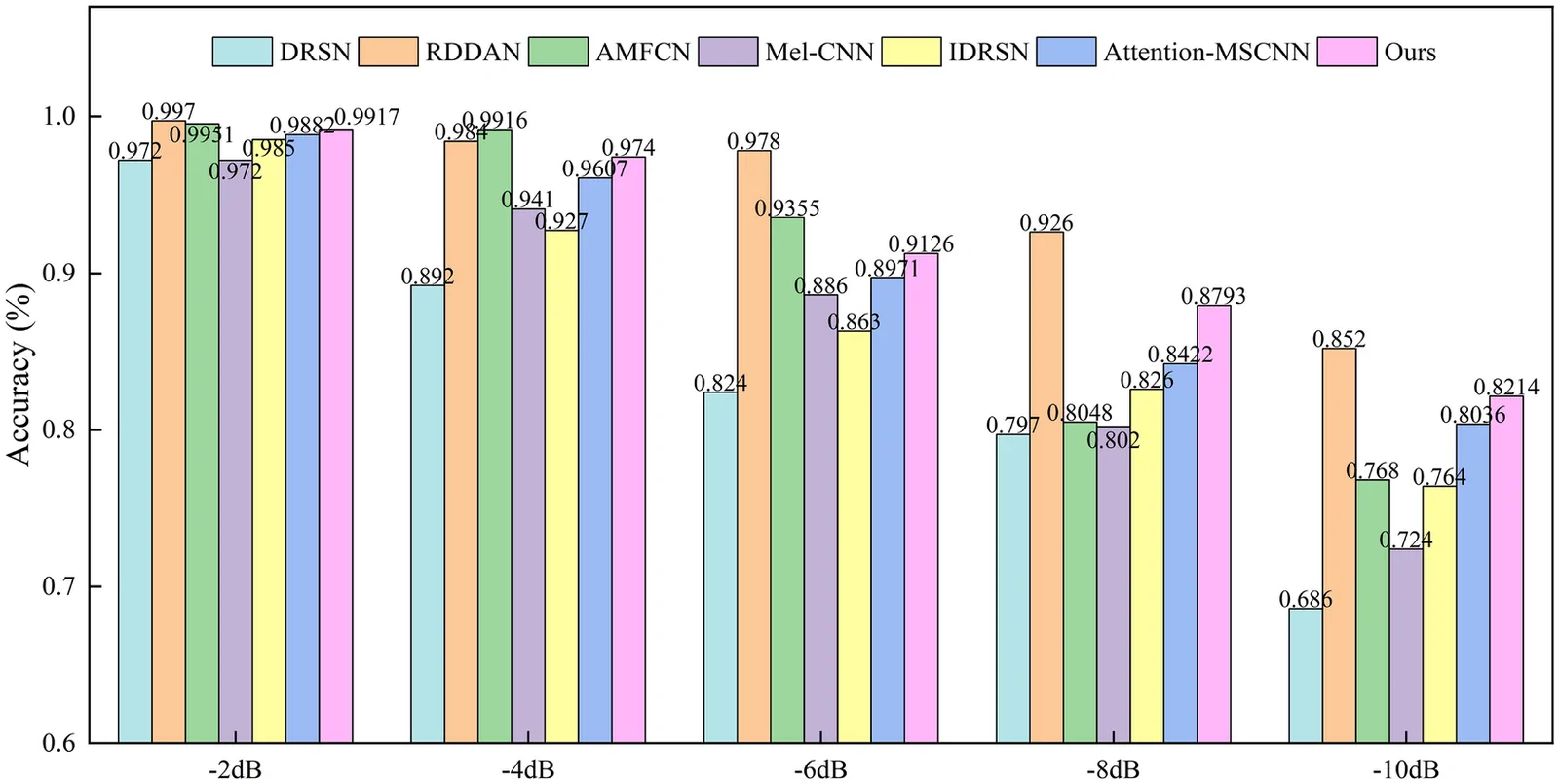
Accuracy comparison of the proposed model and current mainstream anti-noise models on the CWRU dataset under different SNR conditions (– 2 dB to – 10 dB).

To further validate the noise resistance of the proposed model, a comparative study is conducted on the CWRU dataset against several current mainstream anti-noise bearing fault diagnosis models (DRSN^27^, RDDAN^30^, AMFCN^31^, Mel-CNN^23^, IDRSN^33^, and Attention-MSCNN^34^). The results are summarized in Fig. 7. It is essential to highlight that all the models included in the comparison utilise the results presented in the original publications.

As can be observed, at high to extreme noise levels (– 8 dB, – 10 dB), the diagnostic accuracy of our model is 87.93% and 82.14%, respectively, which is significantly higher than that of DRSN, AMFCN, Mel-CNN, IDRSN, and Attention-MSCNN, and is only surpassed by RDDAN. The reason behind this phenomenon is that the“front-end adaptive signal purification”and“back-end temporal-spectral dual-stream diagnosis framework”can provide double support for the proposed model. When noise is extremely intense, the adaptive mode decomposition module effectively enhances the input signal’s SNR, while the temporal-spectral dual-stream framework can reinforce fault patterns from partially corrupted features by fusing dual-perspective information, thereby avoiding a drastic performance drop. Under moderate noise levels (– 2 dB, – 4 dB, – 6 dB), our model’s accuracy of 99.17%, 97.40%, and 91.26% still outperforms most of the compared models. However, it is lower than RDDAN and, in some SNR settings, also lower than AMFCN. This suggests that some competing methods may achieve comparable or even higher accuracy under certain noise levels. A possible reason is that different models emphasize different aspects of feature learning and noise suppression. For example, methods such as RDDAN combine residual denoising with domain adaptation, which may offer additional advantages when the feature distribution shift is relatively moderate, while AMFCN relies on multi-scale convolutional feature extraction and random sampling strategies, which may be more effective at capturing local discriminative patterns under some intermediate-noise conditions. By contrast, the proposed model is designed to improve the overall diagnostic reliability across a wide noise range through the joint effect of adaptive signal purification and temporal-spectral complementary modeling. Therefore, its superiority is more prominently reflected in the strong-noise regime, where maintaining stable fault-identification capability is more challenging and practically more meaningful. This indicates that the proposed model does not sacrifice“performance under low noise”to gain“performance under strong noise”, but rather maintains good stability across different noise levels.

### Ablation study

To evaluate the contribution of each core component to the final performance of the proposed model, an ablation study involving four model variants was designed and tested on the CWRU dataset:

**Model 1:** Employs only the temporal-spectral dual-stream diagnosis framework without the serial local-global feature extractor and without the adaptive mode decomposition module.

**Model 2:** Adds the serial local-global feature extractor to Model 1.

**Model 3:** Adds the adaptive mode decomposition module to Model 1.

**Model 4:** The proposed full model (adaptive mode decomposition module + temporal-spectral dual-stream diagnosis framework + serial local-global feature extractor).

**Table 4:** Effect of core components on the Accuracy, Precision, Recall, and F1-Score of the proposed model on the CWRU dataset under different SNR conditions (– 2 dB to – 10 dB).

Model	SNR	Accuracy (%)	Precision (%)	Recall (%)	F1-Score (%)
Model 1	– 2 dB	93.22±0.87%	93.14±0.91%	93.47±0.83%	92.84±0.96%
	– 4 dB	91.28±1.13%	91.19±1.08%	91.53±1.17%	90.88±1.21%
	– 6 dB	86.92±1.48%	86.81±1.52%	87.14±1.41%	86.51±1.59%
	– 8 dB	79.25±1.96%	79.11±2.03%	79.58±1.89%	78.68±2.11%
	– 10 dB	70.41±2.51%	70.23±2.47%	70.89±2.56%	69.62±2.63%
Model 2	– 2 dB	95.42±0.71%	95.51±0.68%	95.68±0.74%	95.36±0.79%
	– 4 dB	93.17±0.92%	93.28±0.96%	93.41±0.89%	93.17±1.01%
	– 6 dB	88.85±1.29%	88.76±1.34%	89.12±1.23%	88.43±1.38%
	– 8 dB	84.57±1.61%	84.49±1.57%	84.81±1.68%	84.18±1.73%
	– 10 dB	76.75±2.14%	76.62±2.21%	77.03±2.08%	76.24±2.29%
Model 3	– 2 dB	96.64±0.59%	96.71±0.63%	96.82±0.56%	96.61±0.67%
	– 4 dB	95.56±0.78%	95.63±0.81%	95.79±0.74%	95.49±0.86%
	– 6 dB	90.14±1.07%	90.08±1.12%	90.34±1.01%	89.83±1.18%
	– 8 dB	86.42±1.36%	86.31±1.41%	86.67±1.29%	85.98±1.48%
	– 10 dB	79.67±1.83%	79.54±1.87%	79.91±1.76%	79.21±1.94%
Model 4	– 2 dB	99.17±0.41%	99.23±0.38%	99.31±0.42%	99.14±0.47%
	– 4 dB	97.40±0.58%	97.52±0.62%	97.61±0.55%	97.43±0.61%
	– 6 dB	91.26±0.89%	91.18±0.93%	91.47±0.87%	90.89±0.96%
	– 8 dB	87.93±1.14%	87.81±1.21%	88.12±1.09%	87.54±1.27%
	– 10 dB	82.14±1.46%	82.07±1.52%	82.38±1.41%	81.79±1.58%

As observed from Table 4, under all SNR conditions, Model 2 and Model 3 consistently outperform Model 1 in terms of Accuracy, Precision, Recall, and F1-score, demonstrating the individual effectiveness of both the serial local-global feature extractor and the adaptive mode decomposition module. Specifically, the performance improvement of Model 2 is attributed to the serial local-global feature extractor enhancing the joint modeling of local details and long-range dependencies for time series data. In contrast, the gain achieved by Model 3 is owing to the adaptive mode decomposition module effectively suppressing noise interference and strengthening fault-related components in the front-end stage, thus providing a more learnable signal representation for the subsequent temporal-spectral dual-stream diagnosis framework.

Furthermore, Model 4 achieves the best performance under all SNR conditions. It not only attains the highest accuracy, but also consistently yields the best Precision, Recall, and F1-score values, while exhibiting relatively smaller standard deviations overall. This indicates that the complete model provides not only stronger diagnostic capability but also better stability and robustness. For example, under the extreme noise condition of -10 dB, the Accuracy, Precision, Recall, and F1-score of Model 1 are 70.41±2.51%, 70.23±2.47%, 70.89±2.56%, and 69.62±2.63%, respectively. In comparison, these metrics increase to 76.75±2.14%, 76.62±2.21%, 77.03±2.08%, and 76.24±2.29% for Model 2, to 79.67±1.83%, 79.54±1.87%, 79.91±1.76%, and 79.21±1.94% for Model 3, and finally to 82.14±1.46%, 82.07±1.52%, 82.38±1.41%, and 81.79±1.58% for Model 4. The reason is that the serial local-global feature extractor and the adaptive mode decomposition module can cooperate with each other, further strengthening the fault diagnosis capability of the model in noisy environments.

Moreover, under the -10 dB extreme noise condition, Model 3 shows more pronounced advantages over Model 2 in Accuracy, Precision, Recall, and F1-score. For instance, the accuracy improvements over Model 1 are 9.26 and 6.34 percentage points for Model 3 and Model 2, respectively. This result suggests that when the input signal is severely contaminated by noise, the front-end adaptive mode decomposition module plays a more critical role. By enhancing fault-related components and suppressing irrelevant noise before deep feature extraction, it provides a more reliable foundation for the subsequent temporal-spectral dual-stream network, and is therefore particularly important for improving diagnostic performance under severe noise conditions.

## Limitations and future work

Although the proposed temporal-spectral dual-stream anti-noise bearing fault diagnosis model based on adaptive mode decomposition achieves excellent diagnostic performance under various noise intensities, several limitations still exist.

**Computational cost and feasibility for online deployment:** The proposed model consists of the adaptive mode decomposition module and the temporal-spectral dual-stream bearing fault diagnosis framework, which results in relatively high computational complexity. For offline fault diagnosis or non-real-time analysis scenarios, this computational cost is acceptable. However, if the model is to be deployed in online monitoring systems, especially on resource-constrained edge computing devices, the current computational efficiency may struggle to meet stringent real-time requirements. To enable online deployment, future work will focus on lightweight model optimization from two aspects: exploring more efficient signal decomposition algorithms (e.g., fast VMD variants or empirical wavelet transform); adopting techniques such as model pruning, knowledge distillation, or depthwise separable convolutions to reduce the computational burden of the temporal-spectral dual-stream bearing fault diagnosis framework.

**Limitations of noise environment settings:** The performance evaluation of the proposed model relies solely on additive Gaussian white noise (AWGN) to simulate background noise. Although AWGN is widely used as a benchmark noise for testing, real-world industrial environments often exhibit non-Gaussian noise (e.g., impulsive noise caused by mechanical impacts and colored noise generated by electromagnetic interference). These noise patterns are usually more complex and may exhibit nonlinear coupling with fault features in the time domain or frequency domain, thereby posing greater challenges to the anti-interference capability of the model. Therefore, the performance of the proposed model under real industrial noise conditions remains to be further validated. In the future, we will introduce noise types that better reflect real-world conditions (e.g., impulsive noise, pink noise, or real background noise collected from industrial sites) to test the proposed model. Meanwhile, we will investigate the sensitivity of the model to variations in noise statistical properties and explore joint optimization strategies that integrate the adaptive denoising front-end with the back-end deep network to improve the generalization ability of the model under complex and unknown noise conditions.

In summary, future work should focus on two main aspects: improving the computational efficiency of the proposed model to meet the requirements of online deployment; exploring joint optimization strategies of front-end denoising with back-end intelligent recognition networks to enhance the model’s generalization capability under real industrial noise conditions.

## Conclusion

In response to the current anti-noise models lacking effective front-end noise suppression mechanisms and focusing on the extraction and analysis of time domain features, a temporal-spectral dual-stream anti-noise bearing fault diagnosis model based on adaptive mode decomposition is proposed. Specifically, an adaptive mode decomposition module is first designed to decompose and reconstruct raw noisy vibration signals, suppressing noise and enhancing fault-related components at the signal level. Second, a time-frequency dual-stream bearing fault diagnosis framework is constructed. It mines“complementary representations of the same source signal from different perspectives”through parallel dual paths and fuses them at the feature level, thereby strengthening the model’s fault identification accuracy and robustness under noisy backgrounds. Finally, considering that both time domain and frequency domain sequences are essentially“one-dimensional sequences”, a novel serial local-global feature extractor is developed to perform local feature extraction and global temporal modeling on the time domain or frequency domain representations. Experimental results on two public datasets, CWRU and MFPT, demonstrate that under a wide range of noise interference from -2 dB to -10 dB, the proposed model maintains stable and high diagnostic accuracy on both datasets. Particularly under the extreme condition of -10 dB, the diagnostic accuracy on the CWRU dataset remains at 82.14%, significantly outperforming current mainstream anti-noise models, effectively proving its noise resistance capability.

## Data Availability

The datasets used and/or analysed during the current study available from the corresponding author on reasonable request.
